# Association between training experience and readiness for advance care planning among healthcare professionals: a cross-sectional study

**DOI:** 10.1186/s12909-020-02347-3

**Published:** 2020-11-23

**Authors:** Helen Yue-lai Chan, Annie Oi-ling Kwok, Kwok-keung Yuen, Derrick Kit-sing Au, Jacqueline Kwan-yuk Yuen

**Affiliations:** 1 The Nethersole School of Nursing, Faculty of Medicine, The Chinese University of Hong Kong, 7/F. Esther Lee Building, Hong Kong SAR, China; 2 Department of Medicine and Geriatrics, Caritas Medical Centre, Hong Kong SAR, China; 3 Department of Clinical Oncology, Queen Mary Hospital, Hong Kong SAR, China; 4 CUHK Centre for Bioethics, The Chinese University of Hong Kong, Hong Kong SAR, China; 5 Department of Medicine, Li Ka Shing Faculty of Medicine, The University of Hong Kong, Hong Kong, China

**Keywords:** Advance care planning, Professional, Attitude, Training, Education, Confidence

## Abstract

**Background:**

Training has been found effective in improving healthcare professionals’ knowledge, confidence, and skills in conducting advance care planning (ACP). However, the association between training and its actual practice in the clinical setting has not been well demonstrated. To fill this gap, this paper examines the association between their readiness for ACP, in terms of perceived relevancy of ACP with their clinical work, attitudes toward and confidence and willingness to perform it, based on the Theory Planned Behavior and relevant training experiences.

**Methods:**

An online survey about experiences about ACP of healthcare professionals, including physicians, nurses, social workers, and allied healthcare professionals, currently working in hospital and community care in Hong Kong was conducted.

**Results:**

Of 250 respondents, approximately half (52.0%) had received ACP-related training. Those with relevant training reported significantly more positive in the perceived clinical relevance, willingness, and confidence in conducting ACP and different levels of agreement with 19 out of the 25 statements in a questionnaire about attitudes toward ACP than those without (*p*s ≤ 0.001–0.05). Respondents who received training only in a didactic format reported a significantly lower level of confidence in conducting ACP than did others who received a blended mode of learning (*p* = 0.012). Notwithstanding significant differences between respondents with and without relevant training, respondents generally acknowledged their roles in initiating conversations and appreciated ACP in preventing decisional conflict in surrogate decision-making regardless of their training experience.

**Conclusions:**

This paper revealed the association between training and higher level of readiness toward ACP among healthcare professionals. The findings showed that training is a predictor of their readiness for ACP in terms of perceived relevancy, willingness, and confidence. Those who had received training were less likely to consider commonly reported barriers such as time constraints, cultural taboos, and avoidance among patients and family members as hindrances to ACP implementation.

**Supplementary Information:**

The online version contains supplementary material available at 10.1186/s12909-020-02347-3.

## Background

Advance care planning (ACP) is a communication process for patients to communicate their end-of-life care preferences with family members and healthcare providers [1]. ACP is important for reducing decisional conflict and use of futile treatment and improving documentation of end-of-life care preferences, goal-concordant care and satisfaction with care [2, 3].

Healthcare professionals generally viewed ACP positively and believed that they bear the responsibilities to initiate the conversation, but statistics showed that only around half of them had the experience of ACP conversation with their patients [4–9]. The major challenges being identified were consistent across cultures, including perceived reluctance among patients and families for the conversation, sensitive nature of the conversation, uncertainty in prognostication, inadequate training and skills, and time constraint [5, 10–15].

Certain training interventions have been developed to equip healthcare professionals with the skills to facilitate ACP. Several systematic reviews found that training was effective in improving professionals’ knowledge, confidence, and communication skills in conducting ACP or attitudes towards shared decision-making or end-of-life care although the quality of the evidence was relatively low [16–21]. However, the linkage between the act to initiate ACP and knowledge about or attitude toward ACP in healthcare professionals remained weak [10, 14]. The extent to which the training effect could be translated into care practices is still questionable [22].

Theory of Planned Behavior (TPB) has been used as a theoretical framework to explain the ACP behaviors in nurses [14, 23]. According to the theory, the intention of an individual to perform the target behavior is not merely predicted by his/her attitude towards the behavior (positive or negative appraisal of the behavior), but also subjective norms (perceived social pressure to undertake or not to undertake the behavior) and the level of perceived behavioral control (perceived difficulty of undertaking the behavior) [23]. Zhou and associates (2010) had developed an instrument based on the TPB to assess knowledge, attitudes and behaviors of nurses regarding ACP, but items concerning behavioral intention, subjective norms and perceived control had not been explicitly identified in the measure [14]. Previous studies about healthcare professionals’ perceptions towards ACP only focused on knowledge and attitudes, and the attitudinal changes only concerned about shared decision-making, psychosocial care, and end-of-life care [18, 20]. Core components in the TPB that might explain the ACP behaviors have rarely been studied.

In Hong Kong, the concept of ACP has been introduced in the society for more than a decade [24]. Given the potentially sensitive topics, much concern has been on the public acceptance towards ACP. Local studies generally reported that the public, including patients and older adults, welcomed the chances for expressing their views towards end-of-life care [25–28]. However, ACP has not yet been integrated into the care practices [29]. This situation is expected to be changed since the Hospital Authority which governs the local public healthcare services has recently formulated specific guidelines and templates on ACP to provide clinicians with guidance on its implementation, and the government launched a public consultation on legislation to recognize the legal status advance directives in 2019 due to the growing public awareness [30, 31]. Nonetheless, the current relevant training for healthcare professionals is unstandardized and sporadically organized by various professional societies and non-government organizations, with participation on voluntary basis. Against such a background, it is timely to understand the readiness of the healthcare professionals for conducting ACP using the TPB framework. We conducted a survey primarily aimed to investigate the experiences and attitudes of local healthcare professionals related to ACP. This paper aims to examine the association between training and the readiness of healthcare professionals for conducting ACP, based on secondary analysis of the findings from this survey. Specifically, we compared the core variables in the TPB, including attitudes, subjective norms, perceived control attitudes and behavioral intention, between healthcare professionals who had or had not received training.

## Methods

### Study design

A cross-sectional online survey was conducted between November 2019 and April 2020 in Hong Kong. Given that ACP should be conducted in a team-based approach supported by multidisciplinary, all healthcare professionals, including physicians, nurses, social workers and allied health professionals, involved in direct adult patient care in hospital and community settings were eligible to the study. An online survey method was used because it enabled us to reach a wider group of target population across care settings and to ensure complete anonymity of the participants.

### Instrument

**Fig. 1:**
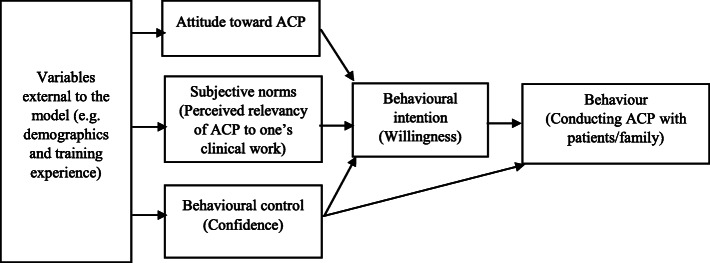
Conceptual framework adapted from Theory of Planned Behavior (Ajzen, 1985)

A questionnaire was developed to investigate healthcare professionals’ experiences and attitudes toward ACP based on a literature review and a team of experts in palliative care and ACP. Demographic data, including age, gender, disciplines, educational level, clinical experience, and current working setting were collected. TPB components in terms of subjective norms, and perceived behavioural control and behavioural intention were framed respectively as perceived relevance of ACP with one’s own clinical work, confidence and willingness for measurement (Fig. 1). Respondents were asked to rate these three aspects on a single-item scale format, from 0 (lowest) to 10 (highest). The three items were “To what extent do you consider ACP related to your current clinical duties?”, “Please rate your level of confidence in conducting ACP with your patients or their family?” and “Please rate your level of willingness in conducting ACP with your patients or their family.” Their attitudes toward ACP were assessed based on their level of agreement with 25 statements related to recommendations for ACP on a five-point Likert scale, from 1 (strongly disagree) to 5 (strongly agree). Participants were also asked about their experience of receiving training related to ACP and whether they had ever conducted ACP with their patients and/or their family members, which is the behaviour concerned in this study.

### Data collection

Ethical approval for the study was obtained from the Survey and Behavioural Research Ethics Committee of the Chinese University of Hong Kong (Ref no.: SBRE-19-112). The link to the online survey was shared through mass emails to potential respondents through personal networks of the researchers as well as social media. Respondents were also encouraged to share the survey with their colleagues and friends. Participation in the study was on voluntary and anonymous bases to ensure privacy.

### Data analysis

Statistical analysis was conducted using SPSS 25.0 (IBM Corp, Armonk, NY). Descriptive statistics was used to summarize the respondents’ characteristics and their responses. The level of agreement with the 25 statements was presented in three levels: strongly disagree/disagree, unsure, and strongly agree/agree, to facilitate analysis. Chi-square test, independent t-test, Mann–Whitney U test, and ANOVA were used to examine the differences in their responses based on training experience. Univariate linear regression was used to identify the association of demographics and training experience with perceived relevancy, willingness, and confidence in conducting ACP. The variables with a *p*-value < 0.01 were included in multiple linear regression for identifying predictors. Variance inflation factors (VIF) were examined to rule out multicollinearity. All statistical tests were two-sided with the level of significance at 0.05.

## Results

### Respondents’ characteristics

A convenience sample of 250 respondents completed the questionnaire (Table 1). Most of them were female (66.4%) and working in public hospital settings (70.7%). Their mean age was 41.8 years (SD 10.3), ranging from 21 to 69. The respondents mainly included physicians (38.8%), nurses (48.8%), and social workers (11.2%), with an average clinical experience of 17.9 years (SD 10.3, in the range 1–42).

**Table 1:** Respondents’ characteristics

	ALL (*N* = 250)	Trained (*n* = 130)	Not trained (*n* = 120)	*p*^Ψ^
Gender				0.165
Male	83 (33.2%)	38 (29.2%)	45 (37.5%)	
Female	167 (66.8%)	92 (70.8%)	75 (62.5%)	
Age (years)^a^	41.8 ± 10.3	43.9 ± 9.19	39.5 ± 11.0	0.001
Disciplines				0.554
Medical doctors	97 (38.8%)	50 (38.5%)	47 (39.2%)	
Nurses	120 (48.0%)	60 (46.2%)	60 (50.0%)	
Allied health	33 (13.2%)	20 (15.4%)	13 (10.8%)	
Clinical experience (years)^#^	17.9 ± 10.3	19.7 ± 9.5	15.9 ± 10.8	0.004
Educational level				0.274
Bachelor	129 (51.6%)	61 (46.9%)	68 (56.7%)	
Master	111 (44.4%)	64 (49.2%)	47 (39.2%)	
Doctoral	10 (4.0%)	5 (3.8%)	5 (4.2%)	
Workplace				0.142
Public hospitals	177 (70.8%)	92 (70.8%)	85 (70.8%)	
Private hospitals	9 (3.6%)	3 (2.3%)	6 (5.0%)	
Community centres	9 (3.6%)	4 (3.1%)	5 (4.2%)	
Care homes	7 (2.8%)	4 (3.1%)	3 (2.5%)	
Hospices	13 (5.2%)	11 (8.5%)	2 (1.7%)	
Private clinics	14 (5.6%)	6 (4.6%)	8 (6.7%)	
Universities	6 (2.4%)	1 (0.8%)	5 (4.2%)	
Others	15 (6.0%)	9 (6.9%)	6 (5.0%)	
Specialty				≤ 0.001
Medical wards	79 (31.6%)	51 (39.2%)	28 (23.3%)	
Long-term	16 (6.4%)	9 (6.9%)	7 (5.8%)	
Community care	20 (8.0%)	12 (9.2%)	8 (6.7%)	
Surgical wards	14 (5.6%)	4 (3.1%)	10 (8.3%)	
Palliative Care	27 (10.8%)	23 (17.7%)	4 (3.3%)	
AED	8 (3.2%)	2 (1.5%)	6 (5.0%)	
ICU/CCU	6 (2.4%)	2 (1.5%)	4 (3.3%)	
Oncology	12 (4.8%)	6 (4.6%)	6 (5.0%)	
O&T	5 (2.0%)	3 (2.3%)	2 (1.7%)	
Psychiatry	19 (7.6%)	5 (3.8%)	14 (11.7%)	
Others	44 (17.6%)	13 (10.0%)	31 (25.8%)	

Footnote: ^Ψ^Chi Square test, unless specified; ^a^M ± SD, independent t test.

### Training experience

Approximately half of the respondents (*n* = 130, 52.0%) had received formal training related to ACP in didactic format only (such as lectures, talks, or seminars) (*n* = 63, 48.5%); a combination of didactic and web-based (*n* = 12, 9.2%); a combination of didactic and workshop (*n* = 29, 22.3%); blended learning with didactic, web-based, and workshop (*n* = 13, 10.0%); and any format with local or overseas placement (*n* = 13, 10.0%). Training was associated with older age (*p* ≤ 0.001), increased years of clinical experience (*p* = 0.004), and working in internal medicine and palliative care specialties (*p* ≤ 0.001).

### Associations between training and readiness for ACP

Table 2 compares the perceived clinical relevance of, and willingness and confidence in, for ACP between respondents who had and had not received relevant training. Respondents who had received training were more likely to find ACP related to their clinical work than the counterparts (*p* ≤ 0.001) and they reported significantly higher levels of willingness (*p* ≤ 0.001) and confidence (*p* ≤ 0.001) with conducting ACP when compared with those who did not receive such training. Univariate linear regression showed that these three variables were associated with specialty and previous ACP training, but not age and clinical experience. Multiple linear regression indicated that respondents received relevant training perceived higher relevancy of ACP in relation to their clinical work (*β* = 0.23, *p* < 0.001), higher level of willingness to conduct ACP with their clients (*β* = 0.30, *p* < 0.001) and higher level of confidence in facilitating the ACP conversation (*β* = 0.35, *p* < 0.001). Specialty is associated with higher level of clinical relevancy (*β* = 0.22, *p* < 0.001) and higher level of confidence (*β* = 0.15, *p* < 0.05), but not for willingness. A significantly higher proportion of respondents who had received ACP training had the experience of conducting ACP with their patients and/or their family members (*p* < 0.001). Table 3 shows that respondents who received blended training generally reported the highest levels of relevance, willingness, and confidence when compared with other modes of learning. Those received training only in didactic format reported the lowest ratings and a significant difference was noted in confidence compared with their counterparts (*p* = 0.012).

**Table 2:** Comparison of readiness for ACP between respondents who had or had not received training (*N* = 250)

	Not trained (*n* = 120)	Trained (*n* = 130)	*p*
Relevancy	6.1 ± 3.3	7.7 ± 2.5	≤ 0.001^a^
Willingness	6.5 ± 2.8	8.2 ± 2.1	≤ 0.001^a^
Confidence	5.3 ± 2.4	7.2 ± 2.2	≤ 0.001^b^
Had experience in conducting ACP with patients and/ or their family	26.7%	75.4%	≤ 0.001^c^

Footnote: ^a^ Independent t test; ^b^ Mann-Whitney U test; ^c^ Chi-square test.

**Table 3:** Comparison of readiness for ACP among respondents who had received different modes of training (*n* = 130)

	Relevancy	Willingness	Confidence
Types of training			
• Didactic format only (n = 63)	7.1 ± 2.7	7.7 ± 2.4	6.6 ± 2.3
• Didactic format and web-based learning (n = 12)	8.3 ± 2.2	8.3 ± 1.3	7.6 ± 1.5
• Didactic format and workshop (*n* = 29)	8.0 ± 1.7	8.7 ± 1.7	8.0 ± 1.3
• Blended learning (*n* = 13)	9.1 ± 1.4	9.2 ± 1.3	8.1 ± 1.7
• Any type with local / overseas placement (n = 13)	7.9 ± 2.9	8.5 ± 2.6	7.4 ± 2.9
*p*	0.068	0.076	0.012

Footnote: ANOVA.

### Comparisons of attitudes toward ACP between trained and non-trained

As shown in Table 4, significant differences were noted between those with and without relevant training in the levels of agreement with 19 out of the 25 statements concerning ACP. Training was associated with perception of more facilitators and lower barriers for ACP. For example, a higher proportion of respondents who had relevant training indicated that they were comfortable with discussing end-of-life care issues with patients and their family members (*ps* ≤ 0.001) than their counterparts. They were more likely to disagree that *“patients and their family members find end-of-life care discussion difficult or a taboo” (ps* ranged from ≤ 0.001– 0.006), but they were less likely to be *“hesitant to follow ACP documents for fear of legal liability”* (*p* ≤ 0.001) and considered time a barrier to conducting ACP (*p* = 0.010*),* compared with those without training*.*

**Table 4:** Comparison of level of agreement regarding ACP  between respondents who had or had not received training (*N* = 250)

	Group	Level of agreement (%)			*p*
		Strongly disagree/ Disagree	Unsure	Strongly agree/ Agree	
**Process**					
ACP should be integrated into routine care services for patients with chronic illness.	Trained	5.4%	11.6%	82.9%	.831
	Not trained	6.7%	13.3%	80.0%	
ACP conversation can be initiated by any health professional.	Trained	13.2%	13.2%	73.6%	.063
	Not trained	17.5%	22.5%	60.0%	
Better not to initiate ACP unless asked by patients or their family members.	Trained	84.5%	7.8%	7.8%	.013*
	Not trained	69.2%	18.3%	12.5%	
ACP should be started early to allow time for contemplation.	Trained	1.6%	11.6%	86.8%	.656
	Not trained	3.3%	11.7%	85.0%	
ACP should not be started before the patients’ condition worsens because their preferences may change according to the context.	Trained	61.2%	14.7%	24.0%	.050*
	Not trained	45.8%	21.7%	32.5%	
ACP is not necessary because use of life-sustaining treatments is a medical decision based on patients’ best interests.	Trained	88.4%	7.0%	4.7%	.072
	Not trained	77.5%	14.2%	8.3%	
Documentation of ACP discussion is useful for care management.	Trained	7.0%	9.3%	83.7%	.052
	Not trained	5.0%	20.0%	75. 0%	
**Outcomes**					
ACP is helpful to clarify patients’ goals and preferences for end-of-life care.	Trained	1.6%	1.6%	96.9%	.193
	Not trained	1.7%	5.8%	92.5%	
ACP destroys patients or their family members’ sense of hope.	Trained	92.2%	1.6%	6.2%	≤.001***
	Not trained	75.0%	15.8%	9.2%	
Under no circumstances should life-sustaining treatments be withheld or withdrawn from patients.	Trained	68.2%	15.5%	16.3%	.014*
	Not trained	50.8%	28.3%	20.8%	
It is hard for patients and/or their family members to reach consensus on end-of-life care.	Trained	43.4%	31.8%	24.8%	≤.001***
	Not trained	21.7%	37.5%	40.8%	
ACP can help to prevent disputes between health care team and family members on medical decisions.	Trained	2.3%	7.8%	89.9%	.036*
	Not trained	3.3%	18.3%	78.3%	
ACP can help to alleviate burden on family decision makers.	Trained	3.1%	5.4%	91.5%	≤.001***
	Not trained	4.2%	21.7%	74.2%	
**Facilitators**					
I am comfortable with discussing end-of-life care issues with patients.	Trained	6.2%	10.9%	82.9%	≤.001***
	Not trained	14.2%	32.5%	53.3%	
I am comfortable with discussing end-of-life care issues with patients’ family members.	Trained	6.2%	11.6%	82.2%	≤.001***
	Not trained	13.3%	30.8%	55.8%	
My seniors/supervisors support me to conduct ACP.	Trained	10.1%	24.8%	65.1%	≤.001***
	Not trained	18.3%	55.8%	25.8%	
My co-workers support me to conduct ACP.	Trained	8.5%	31.0%	60.5%	≤.001***
	Not trained	18.3%	52.5%	29.2%	
The existing ACP policy and guidelines is clear.	Trained	23.3%	28.7%	48.1%	≤.001***
	Not trained	34.2%	50.8%	15.0%	
**Barriers**					
It is difficult to determine if the patient has the mental capacity to make medical decisions.	Trained	54.3%	21.7%	24.0%	.020*
	Not trained	36.7%	28.3%	35.0%	
Patients usually find end-of-life care discussion a taboo.	Trained	46.5%	27.9%	25.6%	≤.001***
	Not trained	23.3%	42.5%	34.2%	
Patients usually find end-of-life care discussion difficult, e.g. difficult to understand the treatments or predict the future.	Trained	42.6%	18.6%	38.8%	.006**
	Not trained	24.2%	30.0%	45.8%	
Patients’ family members usually find end-of-life care discussion a taboo.	Trained	34.9%	27.1%	38.0%	≤.001***
	Not trained	12.5%	29.2%	58.3%	
Patients’ family members usually find end-of-life care discussion difficult, e.g. *difficult to understand the treatments or predict the future.*	Trained	38.0%	14.7%	47.3%	≤.001***
	Not trained	15.0%	31.7%	53.3%	
I am hesitant to follow the preferences stated in the ACP form for fear of legal liability, especially if the patients have not signed an advance directive.	Trained	60.5%	18.6%	20.9%	≤.001***
	Not trained	32.5%	31.7%	35.8%	
I do not have time to conduct ACP.	Trained	43.4%	19.4%	37.2%	.010*
	Not trained	26.7%	32.5%	40.8%	

Footnote: Chi-square test.

By contrast, more respondents who did not have relevant training were uncertain whether *“the existing ACP policy and guidelines are clear*” (*p* ≤ 0.001), whether their *“seniors/supervisors or co-workers support them to conduct ACP*” (*ps* ≤ 0.001), whether “*patients find end-of-life care discussion taboo*” (*p* ≤ 0.001) and the difficulty *“for patients and their family members to reach consensus on end-of-life care*” (*p* ≤ 0.001).

## Discussion

A secondary analysis was conducted to examine the association between healthcare professionals’ experience of ACP training and their readiness for ACP measured in terms of attitudes, subjective norms, perceived behavioral control and behavioral intention. The study findings suggested that the respondents largely have similar views about the merits of ACP, regardless of the training experience. They agreed ACP as instrumental to clarify patients’ preferences and decrease decisional conflicts in surrogate decision-making. High levels of agreement were found on initiating ACP earlier in chronic illness management. The findings were consistent with previous studies that ACP is generally agreed by healthcare professionals as necessary to prepare patients and their families for anticipated difficult decisions and that they play key roles as initiators, educators, and facilitators in ACP [11, 32].

Despite this consensus among the respondents, significant differences were noted in the perceived relevance of ACP with their current clinical work, the level of confidence and willingness to conduct ACP between those who had or had not received relevant training, although training in didactic format appears less promising, and the actual experience of conducting ACP. The findings echoed previous studies that trained healthcare professionals felt more comfortable, willing and confident in end-of-life care communication [18, 19]. Moreover, the findings of this study showed training was the only contributing factor of their behavioural intention, and thereby the actual performance of ACP. Clinical experience of the healthcare professionals did not contribute to their readiness to conduct ACP; and specialty did not contribute to their willingness to undertake the behavior, an important antecedent of performing the behavior.

Another noteworthy result is that, compared with those without training, the trained healthcare professionals were less likely to consider time constraints, cultural taboos, and avoidance among patients and family members as hindering factors to conducting ACP. These concerns have been widely identified as the major barriers to ACP implementation in the literature [10, 11, 13, 14]. Perhaps the skills gained from training had enabled the respondents to approach the topic and facilitate the process tactfully and effectively. Given that ACP should be implemented as a system-wide policy to ensure patients who would like to make plan for their end-of-life care have fair access, ACP training should be provided through blended learning format to all healthcare professionals using an inclusive approach, regardless of specialty or clinical experience, rather than on voluntary basis only to enhance the readiness of the whole healthcare team [29].

### Study strengths and limitations

This paper addresses the knowledge gap about the association between training and healthcare professionals’ attitudes toward ACP. Although the survey included respondents with a wide range of clinical backgrounds, we acknowledged several study limitations when interpreting the study findings. First, the sample was recruited by convenience sampling using an online platform. The respondents might be more interested in ACP-related issues than the non-respondents, and thus the findings could not be generalized due to the potential of participation bias. Second, the causal relationship between training and attitudes toward ACP could not be concluded due to the nature of the study and confounding variables. It is hard to determine if enrollment in training was driven by preceding positive attitude toward ACP. Third, the training experience and attitudes were based on self-reports measured by a self-developed questionnaire. Furthermore, the nature of the training varied greatly in the sample. Robust prospective studies should be conducted to examine the effects of training interventions on the attitudes and actual behaviors related to ACP of healthcare professionals.

## Conclusions

The healthcare professionals’ readiness for conducting ACP and relevant training experience was examined based on secondary analysis of an online survey among healthcare professionals. The findings showed that the trained healthcare professionals perceived higher level of readiness for ACP in terms of clinical relevancy, willingness and confidence, and more positive attitudes toward ACP as they were less likely to consider time constraints, cultural taboos, and avoidance among patients and family members as hindering factors to conducting ACP, compared with those without training.

## Supplementary Information

**Additional file 1.**

## Data Availability

The dataset is available from the corresponding author on reasonable request.

## Abbreviations

ACP: Advance care planning
ANOVA: One-way analysis of variance
M: Mean
SD: Standard deviation
