# Supplementary material for: Association between training experience and readiness for advance care planning among healthcare professionals: a cross-sectional study
Source: BMC Med Educ. 2020 Nov 23;20:451. doi: 10.1186/s12909-020-02347-3 (PMC7684716; doi:10.1186/s12909-020-02347-3)
Supplement: Supplementary file 1 — Additional file 1. [file 12909_2020_2347_MOESM1_ESM.docx]

**Experiences and attitudes of Health Professionals toward Advance Care Planning (ACP)**

| 1. **Demographic** | | | | | | | | | | |  |
| --- | --- | --- | --- | --- | --- | --- | --- | --- | --- | --- | --- |
| 1. Gender | | □ M □ F | | | | | | | | |  |
| 1. Age | |  | | | | | | | | |  |
| 1. Disciplines | | □ Trainees (Years of training: ________)  □ Medical doctors  □ Nurses  □ Social workers | | | | | | | | |  |
| 1. Clinical working experience | | (years) | | | | | | | | |  |
| 1. Highest degree earned | | □ Bachelor degree  □ Master degree  □ Doctoral degree | | | | | | | | |  |
| 1. Current working organisation *(can be more than one option)* | | □ Public hospital  □ Private hospital  □ Neighbourhood Elderly Centre / District Elderly Community centre / Day care centre  □ Residential Care Homes for the Elderly  □ Hospice  □ Private clinic  □ University  □ Others, please specify: __________________ | | | | | | | | |  |
| 1. Current working department *(can be more than one option)* | | □ Medical / Geriatrics  □ Rehabilitation/Infirmary  □ Surgical  □ Palliative Care  □ AED  □ ICU / CCU  □ Oncology  □ Obstetrics / Gynaecology  □ Orthopaedics & Traumatology  □ Psychiatry  □ Others, please specify: __________________ | | | | | | | | |  |
| 1. **Experience with ACP**   Definition of ACP: ACP is an overarching process of proactive communication regarding end-of-life care. Through this process of communication, a patient with advanced progressive disease, his/her health care providers, and his/her family members and caregivers can consider ahead of time what kind of care is appropriate when the patient can no longer make a decision. (Hospital Authority Guidelines on ACP, 2019) | | | | | | | | | | |  |
| 1. To what extent do you consider ACP related to your current clinical duties? | | | | | | | | ______________  (0 – totally irrelevant to 10 – highly relevant) | | |  |
| 1. Please rate your level of willingness in conducting ACP with your patients or their family | | | | | | | | ______________  (0 – lowest to 10 – highest) | | |  |
| 1. Please rate your level of confidence in conducting ACP with your patients or their family | | | | | | | | _____________  (0 – lowest to 10 – highest) | | |  |
| 1. How familiar are you with the Hospital Authority ACP guidelines released in Jun 2019? | | | □ Have read it  □ I know it was released recently but not very familiar  □ Not aware of its existence | | | | | | | |  |
| 1. Have you ever received any formal training related to ACP or end-of-life care discussion? | | | | | | □ Yes 🡪 go to Q5.1  □ No 🡪 skip to Q5 | | | | | |
| - 1. What was the nature of the training? *(can be more than one option)* | | | | | | □ Lecture / Talk / Seminar  □ Online resources  □ Skill building workshop  □ Oversea training  □ Others: ____________ | | | | | |
| - 1. Total hours of training | | | | | | _____ hours | | | | | |
| - 1. What was the focus of the training? *(can be more than one option)* | | | | | | □ Introduction of concept / guidelines  □ Case studies  □ Communication skills  □ Others:_____________ | | | | | |
| 1. Have you ever conducted ACP conversations with your patients and/or their family members? | | | | | | □ Yes 🡪 go to Q6.1  □ No 🡪 skip to next section | | | | | |
| - 1. How frequently did you conduct ACP conversations? | | | | | | □ ≤ once / month  □ 2 – 5 times / month  □ 5 – 10 times / month  □ > 10 times / month | | | | | |
| - 1. Referring to the last time you conducted ACP, why was the discussion arisen? | | | | | | | | | | |  |
|  | □ Diagnosis of a life-limiting condition  □ Early cognitive decline in dementia  □ Significant disease progression *(in terms of functional decline, symptoms burden, deteriorating quality of life)*  □ Discontinuation of disease targeted treatments  □ Transition to palliative care  □ Recovery from an acute severe episode of a chronic disease  □ Following multiple hospital admissions  □ Patient becomes institutionalized  □ Others | | | | | | | | | |  |
| - 1. Who initiated the discussion? | | | □ Doctor  □ Nurse  □ Other health professionals  □ Patient  □ Family members | | | | | | | |  |
| - 1. Who were included in the discussion? *(can be more than one option)* | | | □ Doctor  □ Nurse  □ Other health professionals  □ Patient  □ Family members  □ Others: _________________  □ Not sure | | | | | | | |  |
| - 1. What was the focus of that conversation? *(can be more than one option)* | | | □ Prognosis  □ Patient goals, values and preferences  □ Preferences regarding care preferences near end-of-life  □ End-of-life care/ hospice/palliative care services  □ DNACPR for non-hospitalized patients  □ Withholding or withdrawing of life-threatening treatment(s)  □ Advance directive  □ Others:________________ | | | | | | | |  |
| **The following statements are to understand your attitudes towards ACP. Would you please rate your level of agreement with these statements based on your clinical experience?** | | | | | | | | | | |  |
|  | | | | **1** | **2** | | **3** | | **4** | **5** |  |
|  |  |  |  | **strongly disagree** | **Disagree** | | **Unsure** | | **Agree** | **strongly agree** |  |
| 1. ACP should be integrated into routine care services for patients with chronic illness. | | | |  |  | |  | |  |  |  |
| 1. ACP conversation can be initiated by any health professional. | | | |  |  | |  | |  |  |  |
| 1. Better not to initiate ACP unless asked by patients or their family members. | | | |  |  | |  | |  |  |  |
| 1. ACP is helpful to clarify patients’ goals and preferences for end-of-life care. | | | |  |  | |  | |  |  |  |
| 1. ACP should be started early to allow time for contemplation. | | | |  |  | |  | |  |  |  |
| 1. ACP should not be started before the patients’ condition worsens because their preferences may change according to the context. | | | |  |  | |  | |  |  |  |
| 1. I am comfortable with discussing end-of-life care issues with patients. | | | |  |  | |  | |  |  |  |
| 1. I am comfortable with discussing end-of-life care issues with patients’ family members. | | | |  |  | |  | |  |  |  |
| 1. Under no circumstances should life-sustaining treatments be withheld or withdrawn from patients. | | | |  |  | |  | |  |  |  |
| 1. ACP is not necessary because use of life-sustaining treatments is a medical decision based on patients’ best interests. | | | |  |  | |  | |  |  |  |
| 1. ACP destroys patients or their family members’ sense of hope. | | | |  |  | |  | |  |  |  |
| 1. It is difficult to determine if the patient has the mental capacity to make medical decisions. | | | |  |  | |  | |  |  |  |
| 1. Patients usually find end-of-life care discussion a taboo. | | | |  |  | |  | |  |  |  |
| 1. Patients usually find end-of-life care discussion difficult, *e.g. difficult to understand the treatments or predict the future.* | | | |  |  | |  | |  |  |  |
| 1. Patients’ family members usually find end-of-life care discussion a taboo. | | | |  |  | |  | |  |  |  |
| 1. Patients’ family members usually find end-of-life care discussion difficult, *e.g. difficult to understand the treatments or predict the future.* | | | |  |  | |  | |  |  |  |
| 1. It is hard for patients and/or their family members to reach consensus on end-of-life care. | | | |  |  | |  | |  |  |  |
| 1. ACP can help to alleviate burden on family decision makers. | | | |  |  | |  | |  |  |  |
| 1. ACP can help to prevent disputes between health care team and family members on medical decisions. | | | |  |  | |  | |  |  |  |
| 1. I am hesitant to follow the preferences stated in the ACP form for fear of legal liability, especially if the patients have not signed an advance directive. | | | |  |  | |  | |  |  |  |
| 1. I do not have time to conduct ACP. | | | |  |  | |  | |  |  |  |
| 1. My seniors/supervisors support me to conduct ACP. | | | |  |  | |  | |  |  |  |
| 1. My co-workers support me to conduct ACP. | | | |  |  | |  | |  |  |  |
| 1. The existing ACP policy and guidelines is clear. | | | |  |  | |  | |  |  |  |
| 1. Documentation of ACP discussion is useful for care management. | | | |  |  | |  | |  |  |  |

From your perspective, what other factors / issues may influence your involvement in ACP?

______________________________________________________________________
